# Behavioral Changes in Dogs With Idiopathic Epilepsy Compared to Other Medical Populations

**DOI:** 10.3389/fvets.2019.00396

**Published:** 2019-11-08

**Authors:** Hilary Levitin, Devon Wallis Hague, Kelly C. Ballantyne, Laura E. Selmic

**Affiliations:** ^1^Department of Clinical Veterinary Medicine, University of Illinois College of Veterinary Medicine, Urbana, IL, United States; ^2^Insight Animal Behavior Services PC, Chicago, IL, United States; ^3^Department of Veterinary Clinical Science, The Ohio State University, Columbus, OH, United States

**Keywords:** anxiety, fear, seizure, canine, idiopathic epilepsy

## Abstract

Anxiety related behaviors have been reported in humans diagnosed with idiopathic epilepsy (IE) and such traits may be altered depending on seizure phase. The purpose of this study was to determine the presence and severity of anxiety related behaviors in dogs with IE compared to other medical populations, and to determine if behavioral changes were associated with seizure control. In this retrospective cross-sectional study, the owners of 102 dogs presenting for wellness examination (37), epilepsy (38), and intervertebral disc disease (27) were surveyed utilizing a questionnaire developed based on the shortened Canine Behavioral Assessment and Research Questionnaire (mini-CBARQ), previously validated for its ability to analyze canine behavior. Veterinarians of participating dogs completed a questionnaire to verify diagnoses. Dogs with IE and IVDD had a higher likelihood of being fearful/anxious when approached by an unfamiliar dog compared to the wellness group. Dogs with IE receiving polytherapy had decreased excitement before a walk (*P* = 0.0007) or car trip (*P* = 0.027), increased fear/anxiety when groomed (*P* = 0.0197), and increased shaking, shivering, or trembling when left alone (*P* = 0.0004) compared to dogs receiving monotherapy. Polytherapy dogs had increased agitation when their owner/others showed affection toward other people/dogs during preictal (*P*person = 0.005, *P*animal = 0.0083), postictal (*P*person = 0.001, *P*animal = 0.0068), and interictal (*P*person = 0.0083, *P*animal = 0.02) period compared to monotherapy dogs. Seizure frequency and severity was not correlated with anxiety related behavior in dogs with IE. While seizure phase was associated with behavior changes in 38% (14/37) of our epileptic population, one specific seizure phase was not more likely to produce behavior changes than another. Behavioral changes noted in dogs with IE raises further questions about how this disease affects QoL. Research was presented in abstract form at the ACVIM Forum, Denver, CO, USA, June 2016.

## Introduction

Epilepsy is the most common chronic neurologic disorder in both humans and dogs (1–4). Increasing evidence indicates a correlation between human epileptic seizures and anxiety (5–8). While anxiety has been identified as the most common psychiatric comorbidity in humans with epilepsy, there has also been evidence suggesting that anxiety in adults with epilepsy may be altered depending on the phase of the seizure itself (5–7). Jackson et al. indicates that anxiety can be directly related to the epileptic seizure episode or be present as an interictal symptom. Another study had a similar conclusion, indicating different manifestations of anxiety during the ictal, postictal, and interictal phases of epileptic seizures in adults (5).

In addition to anxiety, fear disorders are common in humans with epilepsy and are often the result of poor seizure control (6). Studies have shown an increase of postictal anxiety and depressive symptoms in people with medically refractory epilepsy or prolonged seizures (5). Seizure semiology has also been proven to be extremely similar in humans and dogs (1, 3, 4, 9–11). It is therefore reasonable to investigate if behavioral changes present in humans with epilepsy are present in dogs.

While anxiety and fear related behaviors are prevalent in dogs (12), Shihab et al. provides the first indication that psychiatric disorders are prevalent in canine patients with idiopathic epilepsy (IE) (4). Behavioral factors like fear or anxiety, defensive aggression, and abnormal perception greatly increased in dogs left untreated for epilepsy, suggesting that there is an association between these factors and the seizure episodes (4). This study provides a solid foundation for the link between anxiety and IE; however, does not differentiate between the different phases of a seizure episode. A recent consensus report defining the seizure phases experienced in veterinary patients revealed that some patients may experience a prodrome prior to the ictal event, which is most commonly exhibited as restlessness, agitation, or attention-seeking; however, this is not reported to be common (13). This consensus report also draws attention to the postictal phase, in which patients may be disoriented or experience other behavioral abnormalities such as vocalization, failure to ambulate with appropriate coordination, aggression, and changes in appetite (13). While it is known that behavior changes may be present in differing variety during seizure phase, to the author's knowledge these changes have yet to be quantified. Closer examination of behavioral changes noted in different phases of a seizure in canine epileptic patients is warranted to determine if a link between those phases and psychiatric comorbidities exist, as it does in humans (5, 6).

The purpose of this study was to determine the presence and severity of anxiety related behaviors in dogs with idiopathic epilepsy when compared to other medical populations, and to determine if behavioral changes present were associated with degree of seizure control. Other medical populations selected for this study were those presenting for wellness examinations or intervertebral disc disease (IVDD). Those in the “wellness” group presented solely for routine wellness examinations to their primary care physicians with the intent of routine vaccination, while those in the “IVDD” group presented for discomfort or neurologic deficits due to intervertebral disc disease diagnosed by a neurologist or surgeon. Findings were determined retrospectively based on a client questionnaire. We hypothesized that anxiety-related behaviors would be more prevalent in the IE group than the IVDD and wellness groups. Within the IE group, we suspect that anxiety-related behaviors will be more pronounced in dogs receiving polytherapy or those with a higher seizure frequency, indicative of poor seizure control.

## Materials and Methods

### Questionnaire Design

A trialed questionnaire (provided as Supplementary Material) designed for this study was based on the Canine Behavioral Assessment and Research Questionnaire (CBARQ) that has been previously validated for its ability to analyze canine behavior (4, 14, 15). We utilized the shortened version, known as the mini-CBARQ, which takes <10 min to complete given that a previous study found that the length of the full CBARQ deterred owners from completing it voluntarily (16). Additionally, a high correlation has been found when comparing results of the full CBARQ to the mini-CBARQ (17). The modified mini-CBARQ questionnaire designed for use in this study was evaluated by a panel of veterinary students along with a board certified behaviorist. Feedback provided was used to modify the questionnaire prior to distribution to ensure ease of comprehension.

### Questionnaire Description

The completed questionnaire was sent to diplomates and candidates in neurology residing in the United States as indicated by the American College of Veterinary Internal Medicine (ACVIM). A total of 286 packets were sent out on the 28th of June, 2013 via the United States Postal Service, each packet containing four copies of the questionnaire. Furthermore, diplomates and candidates of neurology residing in the United States as indicated by the ACVIM received an electronic copy of the questionnaire to increase compliance via the listserv.

Affixed to the owner questionnaire was a cover letter and general information form to be completed. The cover letter stated that by completing this survey, individuals were participating in a study about anxiety and clinical disease status in dogs. It did not indicate our primary focus on behavioral changes related specifically to idiopathic epilepsy so as to avoid receiving biased feedback. The cover letter also stated that the questionnaire should be completed within ~10–15 min to prevent over-thinking and to preserve the most accurate response.

The questionnaire opens with a preliminary section indicating the owner's contact information in addition to their dog's signalment, clinical diagnostic status, current medications, and pertinent diagnostics performed. Epileptic patients receiving one AED were defined as receiving monotherapy and those receiving more than one AED were defined as receiving polytherapy. Seizure frequency was also assessed as an average at the time of diagnosis, and at the current time when the survey was performed. Additionally, it was noted if patients had a normal bile acid, normal magnetic resonance imaging (MRI) of the brain, and/or normal cerebrospinal fluid analysis. Dogs with IE with abnormal neurological examination secondary to anticonvulsants as determined by a neurologist or resident in neurology were also included in this study if upon second examination there were no abnormal neurological findings.

The questionnaire continues with the trialed questionnaire, which was based on the mini- CBARQ. The mini-CBARQ (similar to the full CBARQ) divides behavior into seven sections: “excitability,” “aggression,” “fear and anxiety,” “separation-related behaviors,” “attachment and attention seeking,” “obedience and training,” and “miscellaneous.” The excitability section contains two items evaluating a dog's reaction to exciting events. Reactions range from little or no reaction to over-reaction. The aggression section contains 10 items evaluating different scenarios in which dogs are prone to displaying aggressive behaviors like growling or barking, teeth baring, or biting. The fear and anxiety section contains nine items evaluating different scenarios in which dogs are prone to exhibiting signs of anxiety or fear in response to sounds, objects, people, or specific situation. Signs of anxiety or fear include cowering, retreating, or hiding. The separation-related behavior section includes six items evaluating how dogs react when separated from their owners. These reactions include shaking, salivation, restlessness, vocalization, chewing or scratching, and loss of appetite. The attachment and attention seeking section includes six items evaluating how strongly dogs require the presence and attention of their owner(s). These reactions include following: remaining in close contact with certain household individuals, tendency to nuzzle or paw household individuals, or agitation when certain household members show affection for other people or pets. The obedience and training section includes four items evaluating a dog's ability to obey commands. The miscellaneous section contains 10 items evaluating the general demeanor of individual dogs inside and outside of the house.

All mini-CBARQ items were evaluated using a five-point scale. The excitability, aggression, and fear and anxiety sections were scored 0–4 with 0 indicating that no signs of the behavior were observed in a specific scenario and 4 indicating that the behavior was noted in an extreme fashion. All other sections were scored by identifying how often a behavioral item was exhibited: never, seldom, sometimes, usually, or always.

For owners with epileptic pets, additional information was requested. Owners were asked to estimate general seizure severity based on a ten-point scale of 1 (least severe) to 10 (most severe) as this scale has previously been used to evaluate seizure severity in dogs with idiopathic epilepsy (18). A horizontal line labeled 1 (least severe) to 10 (most severe) was provided and owners were instructed to circle the appropriate number. Sections of the mini-CBARQ relating to separation, attachment and attention seeking, and obedience and training behaviors were modified to include additional parameters for detecting anxiety based behavioral changes associated with different phases of the seizure episode. This part of the questionnaire was only completed by clients with epileptic dogs. These parameters included: “just prior to seizure episode” to indicate a possible prodromal phase, “up to 24 h after seizure episode” to indicate the postictal phase, and “between seizure episodes” to indicate the interictal phase. The terms prodrome, postictal, and interictal were not used on the questionnaire. Instead their definitions as outlined above were used so as to avoid confusion and ensure all owners could provide accurate responses.

Veterinarians treating dogs with idiopathic epilepsy completed a brief questionnaire as well to identify and verify the diagnoses of each dog that participated in this study (provided as Supplementary Material). Veterinarians of dogs with idiopathic epilepsy were additionally asked to indicate when the diagnosis was made, the age of seizure onset, and the results of complete blood count, chemistry profile, serum bile acid testing (if applicable), MRI of the brain, and cerebrospinal fluid analysis.

### Inclusion

Owners of dogs that had been diagnosed with IE between 6 months and 6 years of age were asked to complete the questionnaire. The diagnosis of IE was based on the BMC Veterinary Research correspondence: International veterinary epilepsy task force consensus report on epilepsy definition, classification and terminology in companion animals (13). Dogs with IE were included in this study only if they were found to have seizures with normal hematology as indicated by a complete blood count, normal serum biochemistry profile, and normal interictal neurological examination performed by a neurologist or resident in neurology.

The questionnaire was also distributed to owners presenting dogs of two other medical populations. One group consisted of dogs presenting for annual wellness examination and the other group consisted of dogs presenting for discomfort or neurologic deficits associated with IVDD. Wellness examinations consisted of physical examination by a primary care veterinarian for the sole purpose of vaccination. Intervertebral disc disease was diagnosed by computed tomography (CT) scan or MRI. The dogs composing of these two medical populations did not have a history of seizures. Owners of dogs presenting for IVDD were not asked if decompressive surgery had been indicated or performed in the past, nor was chronicity assessed.

### Statistical Analysis

Dog sex and neuter status, and dog breed were described using frequency and percentages. Dog age was presented as median, minimum, and maximum values given the non-normal distribution as assessed by skewness, kurtosis, normality plots, and Shapiro-Wilk test. The signalment characteristics were statistically compared between populations using Fischer's exact test for dog sex and breed, and Kruskal Wallis test for dog age.

The resulting mini-CBARQ scores of dogs presenting for wellness, IVDD, and those with IE were compared. Fischer's exact tests were utilized, to assess for differences in behavioral traits exhibited between the epileptic, IVDD and wellness groups. If a significant difference between the groups was detected, then *post-hoc* testing with Fischer's exact tests for pairwise comparisons was performed and a Bonferroni correction was applied to reduce the rate of false discovery due to multiple comparisons.

A subgroup analysis was performed for dogs in the epileptic group, this involved assessment for associations between seizure severity, seizure frequency, and seizure therapy (mono- or polytherapy) using Fischer's exact tests. As before, when a significant difference was identified *post-hoc* testing with pairwise comparisons using Fischer's exact tests utilizing a Bonferroni correction was performed. The Bonferroni correction was applied again to reduce the rate of false discovery due to multiple comparison. The effect of seizure phase on behavior was assessed in the epileptic group through generation of new variables with subtraction of one seizure phase behavioral trait score from the other. The new variable non-zero scores reflected a change in behavioral trait score between the two different seizure phases. The scores for the new variable were assessed as frequency and percentage of dogs.

Statistical significance was set at ≤ 0.05 and the statistical analysis was performed using a commercially available software package.

## Results

### Descriptive Data

One hundred and two dogs were included in this study. Thirty dogs met the inclusion criterion for the wellness disease category, 37 dogs met the inclusion criterion for the epilepsy disease category, and 27 met the inclusion criterion for the IVDD category. A summary of signalment characteristics are included in Table 1. The breeds most highly represented in this study include mix (29/102), Dachshund and Miniature Dachshund (6/102 and 3/102, respectively), and Labrador Retriever (6/102).

**Table 1:** Signalment characteristics of study population.

**Variable**	**Wellness**	**Epilepsy**	**IVDD**
**Gender**			
Female spayed	16	16	15
Female intact	1	1	0
Male castrated	20	18	12
Male intact	1	2	0
**Age in years**			
Mean	4.3	5.6	6.7
Range	0.2–12.5	1–13	2–12
**Breeds**			
Mix	11	12	6
Labrador retriever	1	4	1
Golden retriever	1	3	0
Cocker spaniel	0	3	0
Dachshund	0	0	6
Mini dachshund	1	0	3
Other^*^	24	15	11

* *Dogs of other breeds were represented in this study, but their frequencies per disease group were low at 1–2 subjects per breeds. These breeds included: Basset hound, Bloodhound, Border collie, Boston terrier, Boxer, Brussels Griffon, Chesapeake Bay Retriever, Chihuahua, Collie, German Shepherd, Great Dane, Greyhound, Japanese Chin, Miniature Pincher, Miniature Poodle, Neapolitan Mastiff, Old English Sheepdog, Pekingese, Pembroke Welsh Corgi, Pomeranian, Poodle, Pug, Red English Tick Hound, Rottweiler, Rough Coated Collie, Shetland Sheepdog, Toy Poodle, Weimaraner, Welsh Corgi, and Yorkshire Terrier*.

### Epileptic Group

Of the 37 dogs in the epileptic group, 32 (86%) were receiving anticonvulsant therapy. Dogs received one or more of the following anticonvulsant medications to aid in seizure control: phenobarbital (*n* = 21), potassium bromide (*n* = 13), zonisamide (*n* = 7), and levetiracetam (*n* = 8). Within the group of epileptic dogs receiving anticonvulsive therapy, 43% (16/37) were receiving monotherapy, 43% (16/37) were receiving polytherapy, and 14% (5/37) were not receiving an anticonvulsant. A summary of anticonvulsant therapy within the epileptic group can be found in Table 2.

**Table 2:** Characteristics of anticonvulsant therapy in dogs with IE.

**Anticonvulsant**	**Total**	**Monotherapy**	**Polytherapy**
Phenobarbital	21	5	16
Potassium bromide	13	3	10
Zonisamide	7	2	5
Levetiracetam	8	4	4

### Differences in Behavior Between Healthy Dogs, Epileptic Dogs, and Dogs With IVDD

Those dogs in the epilepsy and IVDD groups were more likely to be fearful/anxious when approached by an unfamiliar dog than those dogs in the wellness group (question 17, *P* = 0.0011). No other survey questions assessing other behavioral characteristics were identified as significantly different between the three populations evaluated in this study.

### The Effect of Seizure Severity on Behavior in Epileptic Dogs

The median (range) seizure severity score reported in the epileptic group was 7.0 (range: 2–10) on a scale of 1 (least severe) to 10 (most severe). Increasing seizure frequency was defined as an average number of seizures occurring more frequently at the time of the questionnaire was performed than at the time of initial diagnosis. Epileptic dogs with increasing seizure severity were significantly more likely to show fear and anxiety when approached by an unfamiliar dog (question 17, *P* = 0.0129). No other survey questions were identified as significantly associated with seizure severity.

### The Effect of Seizure Frequency on Behavior in Epileptic Dogs

The median (range) seizure frequency reported in the epileptic group was 4 (range: 1–365) times per year. Epileptic dogs with higher seizure frequency showed significantly less excitement just before being taken on a car trip (question 2, *P* = 0.0126). No other survey questions were identified as significantly associated with seizure frequency.

### The Effect of Treatment (Monotherapy vs. Polytherapy) on Behavior in Epileptic Dogs

Dogs receiving polytherapy have a significant decrease in excitement just before taking a walk and just before being taken on a car trip when compared to dogs on monotherapy (questions 1, *P* = 0.0007 and 2, *P* = 0.0270). Dogs receiving polytherapy have significantly increased fear and anxiety when groomed or bathed by a household member when compared to dogs on monotherapy (question 21, *P* = 0.0197). Dogs receiving polytherapy have a highly significant increase in shaking, shivering, or trembling when left or are about to be left on their own when compared to dogs on monotherapy (question 22, *P* = 0.0004). Dogs receiving polytherapy have a significant increase in becoming agitated when you [their owner] or others show affection for another person, and when you [their owner] or others show affection for another dog or animal when compared to dogs on monotherapy (questions 32, *P*pre = 0.0050, *P*post = 0.0010, *P*inter = 0.0083, and 33, *P*pre = 0.0068, *P*post = 0.0068, *P*inter = 0.0206). No other survey questions were identified as significantly associated with monotherapy compared to polytherapy.

### The Effect of Seizure Phase on Behavior in Epileptic Dogs

A Fisher's exact test was performed on questions pertaining to separation, attachment and attention seeking, and obedience and training behaviors to investigated if an association existed between seizure phase and different behavioral traits. This analysis identified behavior changes across the varying seizure phases in 14 of 37 total epileptic dogs in a majority of questions presented (questions 22–25, 28–31, 34–37). The *P*-values associated with the Fisher's exact tests performed on this section of the questionnaire (questions 22–37) were all statistically significant (*P*pre, *P*post, *P*inter <0.05). This indicates that seizure phase is associated with behavior changes, although one specific seizure phase was not more likely to produce behavior changes than another.

A high level of significance (*P*pre, *P*post, *P*inter < 0.0001) was noted in several behavioral categories of this section of the questionnaire, which included questions relating to separation-related behaviors, attachment/attention seeking, and obedience and training. The behaviors associated with a high level of significance (*P*pre, *P*post, *P*inter < 0.001) included: loss of appetite when left or about to be left alone (question 27); strong affection for one particular member of the household (question 28); tendency to nudge, nuzzle, or paw owner (or others) for attention when their owner is sitting down (question 31); agitation (whines, jumps up, tries to intervene) when their owner (or others) shows affection for another person or another dog (question 32 and 33); obeying the “stay” command (question 35); and exhibiting a high propensity to be distracted by interesting sights, sounds, or smells (question 37).

## Discussion

This study utilized a modified, trialed version of the mini-CBARQ to investigate behavioral changes in dogs with IE. Dogs in the IE and IVDD groups exhibited anxiety related behaviors that were not present in the wellness group. Additionally, differences in behavior were noted between patients with IE receiving polytherapy vs. monotherapy, suggesting that seizure control may influence the behavioral comorbidities. No specific seizure phase was associated with increased anxiety in the present study as it has been seen with humans. The goal of investigating behavioral changes is to better assess QoL, which has become a growing area of interest in the veterinary community.

Dogs with IE and IVDD may have a higher degree of anxiety than dogs presenting for wellness examinations. This was seen as a higher likelihood of dogs with IE and IVDD to be fearful/anxious when approached by an unfamiliar dog. This could be a side effect to the medications, as most of these dogs were on drug therapy for seizure control or pain relief. Or rather, this could be attributed to the disease itself. Dogs with idiopathic epilepsy and IVDD suffer from acute episodes of seizures and pain, respectively. The anticipation of such events may cause them to be fearful/anxious when approached with an unfamiliar situation. Similarly, patients undergoing orthopedic surgery may experience similar anticipatory pain. The use of anxiolytic medications has been investigated for its efficacy in reducing anxiety, agitation, and distress in dogs during the post-operative period following orthopedic surgery, revealing calming, and increased tolerance of confinement in 89% of patients (18). While dogs with IE and IVDD showed evidence of fear and anxiety when approached by an unfamiliar dog, no other parameters were statistically significant in regard to anxiety related behaviors when comparing all groups. It is possible that further correlations were not made between behavioral comorbidities and differing medical populations due to the limited sample size.

Seizure phase has been associated with changes in anxiety related behavior in humans (5, 6). Our cohort of patients showed a similar phenomenon. Behavior changes were noted across the varying seizure phases in 14 of 37 total epileptic dogs in a majority of questions presented (questions 22–25, 28–31, 34–37). Furthermore, a high level of statistical significance was seen between variation of seizure phase and many behavioral changes relating to separation-related behaviors, attachment/attention seeking, and obedience and training (*P*pre, *P*post, *P*inter < 0.001 for questions 27, 28, 31–33, 35, and 37). While it cannot be said that an increase in anxiety was evident in any particular seizure phase, this suggests a difference exists between these phases in dogs with IE which warrants further research on this topic with a larger study population.

Dogs with IE receiving polytherapy exhibit traits that may represent increased generalized anxiety. This subpopulation exhibited less excitement prior to going on a walk or taking a car trip; had increased fear/anxiety when being groomed or bathed by a member of the household; were more likely to shake, shiver, or tremble when left alone or about to be left on their own; and had a higher likelihood of becoming agitated when their owner or others showed affection for another person or another animal. In humans, increased postictal anxiety and depression disorders were found in patients with refractory epilepsy or seizures of prolonged duration (5). Shihab et al. demonstrated that dogs with uncontrolled epilepsy had an increase in behavioral changes, defining uncontrolled as lacking a ≥50% reduction in seizure frequency when receiving a specific combination of antiepileptic drugs: phenobarbital and potassium bromide (4). While a strong connection between the postictal phase and heightened anxiety cannot be made in dogs based on this study [as it was made for humans (5)], there is an increase in anxiety related behaviors in dogs with IE receiving polytherapy as opposed to the monotherapy population.

We hypothesized that increased seizure frequency would be found in the epileptic population receiving polytherapy in this study; however, this was not found to be statistically significant. The only behavior affected by seizure frequency was excitability. Dogs with higher seizure frequency exhibited less excitement before taking a car trip than dogs with lower seizure frequency. Again, this could be a sedative side effect of the antiepileptic medications or possibly due to an overall anticipatory anxiety in these patients.

While anxiety related behaviors have been noted in humans diagnosed with epilepsy (5–8), very few studies have exhibited that anxiety disorders are more common in epileptic dogs than other medical populations. Shihab et al. began the discussion on behavioral changes in dogs with idiopathic epilepsy and provided a strong foundation for this subject. A recent study highlights the presence of attention deficit/hyperactivity disorder-like behaviors in epileptic rodent models and human children and investigated if these types of behaviors were present in dogs as well (19). Packer et al. concluded that dogs with idiopathic epilepsy exhibit certain behaviors that are similar to attention-deficit/hyperactivity disorder (ADHD) symptoms present in human and rodent epileptic patients. The prevalence of ADHD symptoms has also been discussed in review literature pertaining to the impact of IE of QoL (20). To the author's knowledge, no previous study has investigated the relationship between seizure phase, frequency, severity and behavioral comorbidities in canine patients.

The goal of elucidating behavioral changes in dogs with IE is to improve their quality of life (QoL). Wessmann et al. designed a questionnaire directed toward the owners of dogs with IE, with the intent of designing an objective way to approach the subject of QoL to aid both clinicians and dog owners in pertinent decision making such as continued medical management vs. euthanasia (21). In addition, a review article published in 2015 aimed to elucidate this topic and raised concerns for chronic negative effects of epilepsy on our canine patients' quality of life, specifically relating to seizure severity, seizure phase, seizure type, and medication side effects (20). A previous study published in 2006 by Chang more specifically investigated owner perspective on management of dogs with idiopathic epilepsy being treated with phenobarbitone and/or potassium bromide (22). QoL was noted to be one of the three greatest concerns for owners in these patients, along with adequate seizure frequency and acceptable side effects of AEDs (20). Perhaps one of the first studies to investigate owner perception of quality of life performed by Lord and Podell in 1999, and QoL did not appear to be perceived as compromised by pet owners (23). While Lord and Podell did not find a negative impact on patient or their family's QoL, it has been reported more recently that a majority of families of dogs with epilepsy feel that their condition has a negative influence on their daily life due to frequent discussions regarding their dogs' QoL, medication concerns, and the possibility of euthanasia (24). There are certainly adequate inquiries for more information regarding comorbidities in epileptic canine patients, how the epileptic state and these comorbidities affect their day to day behaviors, and how owners perceive these behaviors to affect their pet's QoL.

There are recognized limitations to this retrospective questionnaire-based study. This study included a small sample size, which may have affected the author's ability to produce globally representative findings. The questionnaire was sent only to boarded neurologists, neurology residents, and their patients, which may have biased our population to include the most severe cases of IE and thus might not represent the general population of dogs with IE. Additionally, the questionnaire did not standardize the time frames reported for seizure frequency, leaving this as an open-ended question which could have confounded results as well. For simplicity of study design, distinction was not made in the IVDD group if surgery was indicated or pursued. Surgical therapy would presumably influence not only pain level, but also the medications being administered. Given that medication side effects can include sedation or agitation, more detailed review of medications received would have been indicated to rule out medication side effect as a cause of behavioral changes.

In conclusion, certain anxiety related behaviors were noted more often in dogs with IE receiving polytherapy when compared to those receiving monotherapy. While anxiety related behaviors were not significantly more prominent in dogs with IE compared to other medical populations in this study, this may have been due to limited sample size and warrants further investigation. Future studies involving a larger sample size of both referral and general practice IE patients are indicated to obtain a more global representation of behavioral changes noted in IE patents compared to other medical populations. This may elucidate if anxiolytics might provide benefit in dogs with IE during the postictal or interictal period. Evidence of behavioral changes present throughout different seizure phases within the group of dogs with IE raises further questions about how epilepsy affects canine patient's QoL.

## Data Availability Statement

The raw data supporting the conclusions of this manuscript will be made available by the authors, without undue reservation, to any qualified researcher.

## Ethics Statement

The animal study was reviewed and approved by Institutional Animal Care and Use Committee (IACUC)—University of Illinois. Written informed consent was obtained from the owners for the participation of their animals in this study.

## Author Contributions

The experiment was designed by HL, DH, KB, and LS. The experiment was performed by HL and DH. The data were analyzed by HL, DH, KB, and LS. The paper was written by HL, DH, KB, and LS.

### Conflict of Interest

KB was employed by Insight Animal Behavior Services PC, Chicago, IL, USA. The remaining authors declare that the research was conducted in the absence of any commercial or financial relationships that could be construed as a potential conflict of interest.

## Supplementary Material

The owner questionnaire (11.1) and veterinarian questionnaire (11.2) utilized for this study are provided as Supplementary Material. They can be found online at https://www.frontiersin.org/articles/10.3389/fvets.2019.00396/full#supplementary-material

## Abbreviations

AED: antiepileptic drug
ACVIM: American college of veterinary internal medicine
ADHD: attention-deficit/hyperactivity disorder
CBARQ: canine behavioral assessment and research questionnaire
IE: idiopathic epilepsy
IVDD: intervertebral disc disease
QoL: quality of life.
