# Supplementary material for: Behavioral Changes in Dogs With Idiopathic Epilepsy Compared to Other Medical Populations
Source: Front Vet Sci. 2019 Nov 8;6:396. doi: 10.3389/fvets.2019.00396 (PMC6857470; doi:10.3389/fvets.2019.00396)
Supplement: Supplementary file 1 [file Data_Sheet_1.PDF]

University of Illinois
College of Veterinary Medicine
1008 W Hazelwood Dr
Urbana, IL 61802
Phone: (217) 333-5300
Fax: (217) 244-9554

The University of Illinois College of Veterinary Medicine Neurology Service is compiling information about behavioral tendencies and clinical disease status in dogs.

There is a large pool of literature in human medicine linking behavioral disease in patients with epilepsy. There was a recent study evaluating behavioral changes in dogs associated with the development of primary epilepsy (Shihab, et al. 2012). We are evaluating the behavior in different canine populations - patients with primary epilepsy, patients with intervertebral disc disease, and healthy dogs. Additionally, we are interested to see if there are specific differences in the behaviors in dogs with primary epilepsy in the preictal, postictal, and interictal states.

Please ask your client to fill out the attached questionnaire. Additionally, we ask that you fill out a brief questionnaire regarding the patient's diagnosis and/or provide a copy of the patient's medical record. Return the completed questionnaires by fax (217-244-9554) or scan and email to seizures@vetmed.illinois.edu.

Additionally, we are trying to obtain video of seizures in as many idiopathic epileptic patients as possible. Ideally the video would contain the preictal, ictal and post ictal states of the seizure in order for us to perform specific behavioral analysis. Video recordings of any seizure activity will be helpful. These videos can be emailed directly to seizures@vetmed.illinois.edu.

We understand the time and effort it takes in a busy clinical practice to provide information for our study and your participation will be greatly appreciated. The information that you and your colleagues supply will further our knowledge of behavior in dogs with clinical disease. We thank you for your assistance in this study.

Thank you again for taking the time to complete our questionnaire.

Sincerely,

Devon Wallis Hague, DVM
Clinical Assistant Professor in Neurology at University of Illinois

Kelly Ballantyne, DVM
Behavior Service at Furnetic Chicago Center for Veterinary Medicine

Hilary Levitin
Veterinary Student (Class of 2015)

Veterinarian Use Only

Veterinarian Name:

Patient Name:

Client Name:

Please indicate why the patient presented to you today (circle one):

Wellness exam   Epilepsy/seizures   Intervertebral disc disease (IVDD)

Does the patient have any history of behavioral problems (circle one):   Yes   No

If yes, briefly describe:

If the patient is presenting for <u>IVDD</u>, please indicate how the diagnosis was obtained:

If the patient is presenting for <u>primary epilepsy</u>, please indicate the following when the diagnosis was made:

Age of first seizure:

Neurologic Examination Findings (circle one)?   Normal   Abnormal

If abnormal, briefly describe:

Complete blood count and chemistry profile results (circle one)?   Normal   Abnormal

If abnormal, briefly describe:

Serum bile acid testing (circle one)?   Normal   Abnormal   Not performed

MRI of the brain (circle one)?   Normal   Abnormal   Not performed

If abnormal, briefly describe:

Cerebrospinal fluid analysis (circle one)?   Normal   Abnormal   Not performed

If abnormal, briefly describe:

****Please have your client fill out the remaining questionnaire. Send this form with the owner's completed questionnaire to the University of Illinois via fax at 217-244-9554 or via email**

(seizures@vetmed.illinois.edu).

University of Illinois
College of Veterinary Medicine
1008 W Hazelwood Dr
Urbana, IL 61802
Phone: (217) 333-5300
Fax: (217) 244-9554

The University of Illinois College of Veterinary Medicine's neurology department is compiling data for a study on anxiety and clinical disease status in dogs. You have been chosen to complete this survey to broaden our knowledge of behavioral tendencies in both healthy and clinically ill dogs. The attached survey should take 10-15 minutes to complete. Upon completion, return this packet to your veterinarian. Please take this page home with you for your own records.

If your pet is here for seizures and you have any video recordings prior to, during, and after the seizure episodes, please e-mail it to: **seizures@vetmed.illinois.edu**.

We understand that your time is valuable and greatly appreciate you taking the time to participate in our study. Thank you again for your participation.

Sincerely,

Devon Wallis Hague, DVM
Clinical Assistant Professor in Neurology at University of Illinois

Kelly Ballantyne, DVM
Behavior Service at Furnetic Chicago Center for Veterinary Medicine

Hilary Levitin
Veterinary Student (Class of 2015)

University of Illinois
College of Veterinary Medicine
1008 W Hazelwood Dr
Urbana, IL 61802
Phone: (217) 333-5300
Fax: (217) 244-9554

Client Information

Name: Phone: E-mail:

In the event that clarification is required, would you allow us to contact you? Yes No

Please bear in mind that we will only contact you if one of your answers on this survey requires clarification and for no other reasons.

Pet Information

Name: Breed: Age:

Sex: ☐ Intact Male ☐ Intact Female ☐ Neutered Male ☐ Neutered Female

What is your pet coming in for today? Please circle applicable answer.

☐ Wellness exam ☐ Epilepsy/seizures ☐ Intervertebral disc disease (IVDD)

If your pet is here for epilepsy, when did the seizures begin?

How often did they occur after 1st diagnosed?

How often do they currently occur?

Please rank the severity of your pet's seizures by circling the appropriate number

Dog remains standing during a fit	Dog lies down/falls over during fit
(less severe fits may only involve the head or one side of the dog, more severe fits may involve the whole body)	(more severe fits may involve urination, defecation or complete loss of consciousness)

least severe ***most severe***

1	2	3	4	5	6	7	8	9	10

Please list any and all medications your pet is currently taking in the space below.

If your pet is here for epilepsy, please list any medications previously given and why you switched to current medication(s).

Does your pet experience anxiety or aggressive episodes? Please list any previous **history of behavioral issues** you have experienced with your pet in the recent past.

Please fill out the following questionnaire. If your dog is here for **seizures(epilepsy)**, please check off the gray boxes as well. Again, thank you so much for participating! Your response is greatly appreciated.

Excitability

Some dogs show little reaction to exciting events, while others become highly excited at novelty. Based on a 5-point scale (0 = calm, 4 = extremely excitable), circle the number that indicates your own dogs recent tendency to become excitable in the following situation.

1. Just before taking a walk

		**Mild-Moderate excitability**				
**Calm**: little or no special reaction	0	1	2	3	4	**Extremely excitable:** over-reacts, hard to calm down

2. Just before being taken on a car trip

		**Mild-Moderate excitability**				
**Calm**: little or no special reaction	0	1	2	3	4	**Extremely excitable:** over-reacts, hard to calm down

Aggression

Most dogs display aggressive behaviors occasionally, including barking, growling, baring teeth, snapping, etc. Please circle the appropriate number based on a 5-point scale (0 = no aggression, 4 = serious aggression) to indicate your own dog's recent tendency to display aggressive behaviors in each of the following circumstances.

3. When approached directly by an unfamiliar person while being walked/exercised on a leash

		**Mild-Moderate aggression:** growling/barking - baring teeth				
**No aggression:** No visible signs of aggression	0	1	2	3	4	**Extremely aggression:** Snaps, bites, or attempts to bite

4. When toys, bones or other objects are taken away by a household member

		**Mild-Moderate aggression:** growling/barking - baring teeth				
**No aggression:** No visible signs of aggression	0	1	2	3	4	**Extremely aggression:** Snaps, bites, or attempts to bite

5. When approached directly by a household member while s/he (the dog) is eating

		**Mild-Moderate aggression:** growling/barking - baring teeth				
**No aggression:** No visible signs of aggression	0	1	2	3	4	**Extremely aggression:** Snaps, bites, or attempts to bite

6. When mailmen or other delivery workers approach your home

Mild-Moderate aggression:

**No aggression:**		growling/barking - baring teeth				**Extremely aggression:**
No visible signs of aggression	0	1	2	3	4	Snaps, bites, or attempts to bite

7. When his/her food is taken away by a household member

		**Mild-Moderate aggression:**				
**No aggression:**		growling/barking - baring teeth				**Extremely aggression:**
No visible signs of aggression	0	1	2	3	4	Snaps, bites, or attempts to bite

8. When approached directly by an unfamiliar dog while being walked/exercised on a leash

		**Mild-Moderate aggression:**				
**No aggression:**		growling/barking - baring teeth				**Extremely aggression:**
No visible signs of aggression	0	1	2	3	4	Snaps, bites, or attempts to bite

9. When strangers walk past your home when your dog is outside or in the yard

		**Mild-Moderate aggression:**				
**No aggression:**		growling/barking - baring teeth				**Extremely aggression:**
No visible signs of aggression	0	1	2	3	4	Snaps, bites, or attempts to bite

10. When barked, growled, or lunged at by another (unfamiliar) dog

		**Mild-Moderate aggression:**				
**No aggression:**		growling/barking - baring teeth				**Extremely aggression:**
No visible signs of aggression	0	1	2	3	4	Snaps, bites, or attempts to bite

11. When approached while eating by another (familiar) household dog (leave blank if no other dogs)

		**Mild-Moderate aggression:**				
**No aggression:**		growling/barking - baring teeth				**Extremely aggression:**
No visible signs of aggression	0	1	2	3	4	Snaps, bites, or attempts to bite

12. When approached while playing with/chewing a favorite toy, bone, object, etc., by another (familiar) household dog (leave blank if no other dogs)

		**Mild-Moderate aggression:**				
**No aggression:**		growling/barking - baring teeth				**Extremely aggression:**
No visible signs of aggression	0	1	2	3	4	Snaps, bites, or attempts to bite

Fear and Anxiety

Dogs often show signs of anxiety or fear when exposed to particular sounds, objects, persons or situations, for example: crouching or cringing with tail tucked between legs; whimpering or whining; freezing; trembling; or attempting to escape or hide. Please circle the appropriate number based on a 5-point scale (0 = no fear, 4 = extreme fear) to indicate your own dog's recent tendency to display fearful behavior in the following circumstances.

13. When approached directly by an unfamiliar person while away from your home

			**Mild-Moderate fear/anxiety**			
**No fear/anxiety:**						**Extremely fear:**
No visible signs of fear.	0	1	2	3	4	Cowers, retreats, or hides, etc.

14. In response to sudden or loud noises (e.g. thunder, vacuum cleaner, car backfire, road drills, objects being dropped, etc.)

			**Mild-Moderate fear/anxiety**			
**No fear/anxiety:**						**Extremely fear:**
No visible signs of fear.	0	1	2	3	4	Cowers, retreats, or hides, etc.

15. When an unfamiliar person tried to touch or pet the dog

			**Mild-Moderate fear/anxiety**			
**No fear/anxiety:**						**Extremely fear:**
No visible signs of fear.	0	1	2	3	4	Cowers, retreats, or hides, etc.

16. In response to strange or unfamiliar objects on or near the sidewalk (e.g. plastic trash bags, leaves litter, flags flapping, etc.)

			**Mild-Moderate fear/anxiety**			
**No fear/anxiety:**						**Extremely fear:**
No visible signs of fear.	0	1	2	3	4	Cowers, retreats, or hides, etc.

17. When approached directly by an unfamiliar dog

			**Mild-Moderate fear/anxiety**			
**No fear/anxiety:**						**Extremely fear:**
No visible signs of fear.	0	1	2	3	4	Cowers, retreats, or hides, etc.

18. When first exposed to unfamiliar situations (e.g. first car trip, first time in elevator, first visit to veterinarian, etc.)

			**Mild-Moderate fear/anxiety**			
**No fear/anxiety:**						**Extremely fear:**
No visible signs of fear.	0	1	2	3	4	Cowers, retreats, or hides, etc.

19. When barked, growled, or lunged at by an unfamiliar dog

			**Mild-Moderate fear/anxiety**			
**No fear/anxiety:** No visible signs of fear.	0	1	2	3	4	**Extremely fear:** Cowers, retreats, or hides, etc.

20. When having nails clipped by a household member (leave blank if you do not do this at home)

			**Mild-Moderate fear/anxiety**			
**No fear/anxiety:** No visible signs of fear.	0	1	2	3	4	**Extremely fear:** Cowers, retreats, or hides, etc.

21. When groomed or bathed by a household member (leave blank if you do not do this at home)

			**Mild-Moderate fear/anxiety**			
**No fear/anxiety:** No visible signs of fear.	0	1	2	3	4	**Extremely fear:** Cowers, retreats, or hides, etc.

Separation-Related Behavior

Some dogs show signs of anxiety or abnormal behaviors when left alone, even for short periods of time. Thinking back over the recent past, how often has your dog shown the following signs of separation-related behavior when left, or about to be left, on its own? Check the appropriate boxes.

22. Shaking, shivering, or trembling

	Never	Seldom	Sometimes	Usually	Always
Most of the time					
Just prior to seizure episode					
Up to 24 hours after seizure episode					
Between seizure episodes					

23. Excessive salivation

	Never	Seldom	Sometimes	Usually	Always
Most of the time					
Just prior to seizure episode					
Up to 24 hours after seizure episode					
Between seizure episodes					

24. Restlessness/agitation/pacing

	Never	Seldom	Sometimes	Usually	Always
Most of the time					
Just prior to seizure episode					
Up to 24 hours after seizure episode					
Between seizure episodes					

25. Vocalization: whining, barking, howling

	Never	Seldom	Sometimes	Usually	Always
Most of the time					
Just prior to seizure episode					
Up to 24 hours after seizure episode					
Between seizure episodes					

26. Chewing/scratching at doors, floor, windows, curtains, etc.

	Never	Seldom	Sometimes	Usually	Always
Most of the time					
Just prior to seizure episode					
Up to 24 hours after seizure episode					
Between seizure episodes					

27. Loss of appetite

	Never	Seldom	Sometimes	Usually	Always
Most of the time					
Just prior to seizure episode					
Up to 24 hours after seizure episode					
Between seizure episodes					

Attachment and Attention-seeking

Most dogs are strongly attached to their people, and some demand a great deal of attention and affection from them. Thinking back over the recent past, how often has your dog shown each of the following signs of attachment or attention-seeking? Check the appropriate boxes.

28. Displays a strong attachment for one particular member of the household

	Never	Seldom	Sometimes	Usually	Always
Most of the time					
Just prior to seizure episode					
Up to 24 hours after seizure episode					
Between seizure episodes					

29. Tends to follow you (or other members of the household) about the house, from room to room

	Never	Seldom	Sometimes	Usually	Always
Most of the time					
Just prior to seizure episode					
Up to 24 hours after seizure episode					
Between seizure episodes					

30. Tends to sit close to, or in contact with you (or others) when you are sitting down

	Never	Seldom	Sometimes	Usually	Always
Most of the time					
Just prior to seizure episode					

Up to 24 hours after seizure episode					
Between seizure episodes					

31. Tends to nudge, nuzzle or paw you (or others) for attention when you re sitting down.

	Never	Seldom	Sometimes	Usually	Always
Most of the time					
Just prior to seizure episode					
Up to 24 hours after seizure episode					
Between seizure episodes					

32. Becomes agitated (whines, jumps up, tries to intervene) when you (or others) show affection for another person.

	Never	Seldom	Sometimes	Usually	Always
Most of the time					
Just prior to seizure episode					
Up to 24 hours after seizure episode					
Between seizure episodes					

33. Becomes agitated (whines, jumps up, tries to intervene) when you (or others) show affection for another dog or animal.

	Never	Seldom	Sometimes	Usually	Always
Most of the time					
Just prior to seizure episode					
Up to 24 hours after seizure episode					
Between seizure episodes					

Obedience and Training

Some dogs are more obedient and trainable than others. Please check the appropriate boxes to indicate how trainable or obedient your dog has been in each of the following situations in the recent past.

34. Obeys the "sit" command

	Never	Seldom	Sometimes	Usually	Always	N/A
Most of the time						
Just prior to seizure episode						
Up to 24 hours after seizure episode						
Between seizure episodes						

35. Obeys the "stay" command

	Never	Seldom	Sometimes	Usually	Always	N/A
Most of the time						
Just prior to seizure episode						
Up to 24 hours after seizure episode						
Between seizure episodes						

36. Seems to listen closely to everything you say or do

	Never	Seldom	Sometimes	Usually	Always
Most of the time					
Just prior to seizure episode					
Up to 24 hours after seizure episode					

Between seizure episodes					

37. Easily distracted by interesting sights, sounds, or smells

	Never	Seldom	Sometimes	Usually	Always
Most of the time					
Just prior to seizure episode					
Up to 24 hours after seizure episode					
Between seizure episodes					

Miscellaneous Problems

Dogs display a wide range of miscellaneous behavior problems in addition to those already covered by this questionnaire. Thinking back over the recent past, please indicate how often your dog has shown any of the following behaviors by circling the appropriate answer.

38. Escapes or would escape from home or yard, given the chance

Never Seldom Sometimes Usually Always

39. Chews inappropriate objects

Never Seldom Sometimes Usually Always

40. Pulls excessively hard when on leash

Never Seldom Sometimes Usually Always

41. Urinates against objects/furnishings in your home

Never Seldom Sometimes Usually Always

42. Urinates when left alone at night or during the day

Never Seldom Sometimes Usually Always

43. Hyperactive, restless, has trouble settling down

Never Seldom Sometimes Usually Always

44. Playful, puppyish, boisterous

Never Seldom Sometimes Usually Always

45. Active, energetic, always on the go

Never Seldom Sometimes Usually Always

46. Chases own tail/hind end

Never Seldom Sometimes Usually Always

47. Barks persistently when alarmed or excited

Never Seldom Sometimes Usually Always

You have reached the end of our survey. Your participation is greatly appreciated! Please return this packet to your veterinarian.
